# Multiple modifiable maternal, household and health service factors are associated with maternal nutrition and early breastfeeding practices in Burkina Faso

**DOI:** 10.1111/mcn.13457

**Published:** 2022-11-14

**Authors:** Sunny S. Kim, Césaire T. Ouédraogo, Rock R. Zagré, Rasmané Ganaba, Maurice G. Zafimanjaka, Manisha Tharaney, Purnima Menon

**Affiliations:** ^1^ Poverty, Health and Nutrition Division International Food Policy Research Institute (IFPRI) Washington District of Columbia USA; ^2^ Independent Consultant Ouagadougou Burkina Faso; ^3^ Poverty, Health and Nutrition Division IFPRI Dakar Senegal; ^4^ AFRICSanté Bobo Dioulasso Burkina Faso; ^5^ FHI Solutions Ouagadougou Burkina Faso; ^6^ FHI Solutions Washington District of Columbia USA; ^7^ Poverty, Health and Nutrition Division IFPRI New Delhi India

**Keywords:** breastfeeding, Burkina Faso, dietary diversity, iron and folic acid, maternal nutrition

## Abstract

Low coverage of effective nutrition interventions in many high‐burden countries, due to service provision and demand factors, result in poor uptake of recommended practices and nutrition outcomes. We examined the factors that influence maternal nutrition and early breastfeeding practices and determined the extent that the key factors could improve these practices in two regions in Burkina Faso. We used household survey data among pregnant (*n* = 920) and recently delivered women (*n* = 1840). Multivariable regression analyses were conducted to identify the determinants of a diverse diet and iron‐folic acid (IFA) supplement consumption, weight monitoring during pregnancy and early initiation of breastfeeding (EIBF). Population attributable risk analysis was used to estimate how much the outcomes can be improved under optimal conditions of interventions that address the modifiable determinants. During pregnancy, 21% of women achieved minimum diet diversity (MDD‐W), 70% consumed 90+ IFA tablets and 65% were weighed 4+ times; EIBF was 40%. Nutrition knowledge was associated with MDD‐W (odds ratio [OR]: 3.2), 90+ IFA (OR: 1.5) and EIBF (OR: 1.9). Positive social norms and family support were associated with 90+ IFA (OR: 1.5). Early and 4+ ANC visits were associated with 90+ IFA (OR: 1.5 and 10) and 4+ weight monitoring (OR: 6.2). Nutrition counselling was associated with 90+ IFA (OR: 2.5) and EIBF (OR: 1.5). Under optimal programme conditions, 41% of women would achieve MDD‐W, 93% would consume 90+ IFA, 93% would be weighed 4+ times and 57% would practice EIBF. Strengthening the delivery and uptake of interventions targeted at these modifiable factors has the potential to improve maternal nutrition practices.

## Key messages

- In our Burkina Faso study, maternal nutrition practices were low to moderate, with 21% of pregnant women consuming at least 5 food groups, 70% of women consuming at least 90 daily iron and folic acid (IFA) tablets and 65% weighed at least 4 times during their last pregnancy, and 40% of women initiating breastfeeding within 1 h after birth.
- Various individual, household and health service level factors were modifiable through interventions for improving maternal nutrition and breastfeeding practices, but practices were associated with different combinations of factors.
- Under optimal conditions of interventions that address the modifiable determinants, we estimated that nearly all women would be able to achieve consumption of 90+ IFA tablets and receipt of weight monitoring 4+ times, and nearly half or more of the women would be able to achieve minimum dietary diversity and early initiation of breastfeeding.

## Graphical abstract

We examined the factors that influence maternal nutrition and early breastfeeding practices and determined the extent that the key factors could improve these practices in two regions in Burkina Faso. Strengthening the delivery and uptake of interventions targeted at the modifiable factors has the potential to improve nutrition practices.

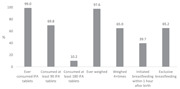

**Abbreviations**
ANC: antenatal care
A&T: Alive & Thrive
EBF: exclusive breastfeeding
EIBF: early initiation of breastfeeding
IFA: iron and folic acid
MDD‐W: minimum dietary diversity for women

## 1 INTRODUCTION

Adequate maternal nutrition is important for maternal health and wellness and is a major determinant of pregnancy outcomes. Maternal malnutrition increases the risk of obstructed labour, preterm delivery or low birthweight, anaemia, hypertension and mortality for the mother (Black et al., 2013; Dean et al., 2014; Ramakrishnan et al., 2012). For the baby, low birthweight increases the risk of mortality, and those who survive are likely to suffer long‐term consequences including growth retardation, lower educational achievement, delivery of smaller babies and lower socioeconomic status in adulthood. (King, 2016; Victora et al., 2008). Recognizing the importance of perinatal nutrition, the World Health Organization (WHO) recommended key nutrition interventions such as nutrition counselling and iron and folic acid (IFA) supplementation as essential components of antenatal care (ANC) (World Health Organization, 2016).

Despite global recommendations, coverage of effective nutrition interventions such as IFA supplementation and counselling on adequate nutrition during pregnancy and optimal breastfeeding during ANC remains low in many high‐burden countries (Heidkamp et al., 2020), due to factors related to supply and service provision as well as service demand and utilization. Poor service quality, inadequate access and negative behavioural determinants among clients (e.g., poor knowledge, attitude, spouse or family support, relationship with a service provider, etc.) may all contribute to low service use.

Various studies and reviews have examined factors affecting the utilization of ANC in developing countries (Aziz Ali et al., 2018; Simkhada et al., 2008), but there is less evidence on factors influencing the use of specific services such as maternal nutrition interventions. A study in Bangladesh observed that early and more ANC visits, receipt of free supplements, good nutrition knowledge and husband's support were associated with higher IFA and calcium consumption (Nguyen et al., 2017). Women were more likely to consume a diverse diet when they had good knowledge, self‐efficacy, positive social norms and husband support (Nguyen et al., 2017). In India, nutrition knowledge, enabling beliefs and self‐efficacy were associated with higher IFA and calcium consumption; nutrition knowledge was also associated with the consumption of a diverse diet and weight monitoring at least 3 times during pregnancy (Nguyen et al., 2019). Thus, there is a growing body of evidence on supply‐ and demand‐side factors that affect maternal nutrition service use and practices.

In Burkina Faso, despite improvement in several health and nutrition indicators, 58% of pregnant women are anaemic (INSD & ICF International, 2012) with about 56% of women having consumed IFA tablets for ≥90 days during their most recent pregnancy, and only half of the women initiated breastfeeding within the first hour of delivery (PMA2020, 2018). Among women of reproductive age who had a live birth in the last 2 years, about 49% were counselled about adequate diet during ANC, and among those who were weighed, only 48% received counselling about adequate weight gain during pregnancy (PMA2020, 2018). There is scarce information on how well maternal nutrition interventions are integrated into ANC services and factors related to their use or maternal nutrition practices in Burkina Faso, although few studies have examined factors related to overall ANC services. A study on the quality of ANC in Gnagna province in the Eastern region observed low skills and ability among service providers at primary health centres as a factor that limited maternal health service use (Nikiema et al., 2010). A qualitative study in five health centres in the Central‐East and Eastern regions observed that household decision‐making structure and family support were important factors in maternal care‐seeking (Somé et al., 2013).

This paper examines the health service, household and maternal factors that influence three maternal nutrition practices (consumption of a diverse diet, consumption of IFA supplements and weight monitoring during pregnancy) and early initiation of breastfeeding (EIBF) and determines the extent that the key factors, if strengthened, could improve these practices in two regions in Burkina Faso.

## 2 METHODS

### 2.1 Programme description

Alive & Thrive (A&T) is an initiative that supports the scaling up of nutrition interventions to save lives, prevent illnesses and contribute to healthy growth and development through improved maternal nutrition and infant and young child feeding practices in several countries. In Burkina Faso, A&T developed a set of interventions aimed at strengthening maternal nutrition services integrated into ANC provided through the government health system (Sanghvi et al., 2022). A&T aimed to test the feasibility of improving the provision and uptake of maternal nutrition interventions such as counselling on dietary diversity, adequate food intake, consumption of IFA supplements, adequate weight gain and early breastfeeding practices and community mobilization.

### 2.2 Data and study population

This study used data from the baseline household survey conducted as part of the evaluation of A&T maternal nutrition interventions in two regions—Hauts‐Bassins and Boucle du Mouhoun, in Burkina Faso (registered at ClinicalTrials.Gov: NCT04155437). These two regions were selected with the Government of Burkina Faso, based on regional‐level engagement and ownership, presence of a cadre of community health workers, size of region and level of security. The survey was carried out in 80 health centre catchment areas in four health districts (Boromo, Toma, Dandé and Léna). Three villages were randomly selected within each health centre catchment area, and a census was conducted within each village; from the two census lists (for pregnant women and recently delivered women), women were selected by simple random sampling until the required sample sizes were reached. The sample included 920 pregnant women and 1840 recently delivered women with children under 6 months of age, to determine current dietary practices during pregnancy and service exposure throughout the last pregnancy and maternal nutrition and early breastfeeding practices respectively.

Structured questionnaires were administrated face‐to‐face using computer‐assisted personal interviewing at the respondents' homes by survey teams trained and supervised under AFRICSanté (Agence de Formation, de Recherche et d'Expertise en Santé pour l'Afrique) in November–December 2019.

### 2.3 Dependent variables

Four primary outcomes related to maternal nutrition practices were constructed: (1) minimum dietary diversity during pregnancy (five or more food groups); (2) consumption of at least 90 IFA tablets during the last pregnancy; (3) weight monitoring at least 4 times during the last pregnancy and (4) EIBF. A fifth outcome of exclusive breastfeeding (EBF) was also analyzed and presented in Supporting Information. Maternal dietary diversity during pregnancy was assessed among pregnant women using the individual report of foods consumed over a 24‐h recall period. These foods were grouped into 10 categories based on the minimum dietary diversity guidelines for women (MDD‐W) (FAO and FHI 360, 2016). A diet diversity score was calculated as the number of food groups consumed out of 10 total food groups, and the cut‐off of at least 5 food groups per day was used to define MDD‐W to achieve micronutrient needs. For the other outcomes, recently delivered women were asked and probed about the number of IFA tablets they consumed and the number of times they were weighed during their last pregnancy. For breastfeeding, women were asked how many hours/days after birth they started breastfeeding their child, and EIBF was defined as within 1 h after birth. EBF was assessed using a report of any foods or liquids fed to the child over a 24‐h recall period and defined as feeding the child no food or liquids other than breast milk in the past 24 h (WHO & UNICEF, 2021).

### 2.4 Independent variables

The independent variables were identified based on a conceptual framework applied in a previous study where the predicted effects of the potentially modifiable determinants were assessed on IFA supplement consumption and dietary diversity outcomes (Nguyen et al., 2017). These determinants included maternal, household and health service factors.

At the maternal level, knowledge scores were generated for dietary diversity, IFA, weight gain during pregnancy and breastfeeding. For knowledge of dietary diversity, women were asked to name at least five food groups, examples of locally available food rich in essential nutrients and the importance of food variety during pregnancy (eight question items). For IFA knowledge, recently delivered women were asked whether they heard of anaemia, its effects and causes, about the recommended numbers of IFA tablets per month and throughout pregnancy and benefits of IFA during pregnancy (5 question items). Knowledge of weight gain was assessed based on women's knowledge of how much weight a pregnant woman should gain during pregnancy, where a response of 10–12 kg was scored as 1, that is, correct. For the knowledge of breastfeeding, women were asked the time after birth a baby should start breastfeeding, the reason a baby should breastfeed soon after birth, the benefits of colostrum, how long a baby should be exclusively breastfed and why and at what age a baby can receive liquid other than breast milk (16 question items). Each question item was scored as 0 or 1, and the sum represented the knowledge score. Belief, self‐efficacy and social norms related to maternal nutrition practices were measured on a 5‐point Likert scale by asking women about the extent to which they agreed or disagreed with statements. Belief and self‐efficacy statements asked women whether they believed that the recommended practices were beneficial and feasible to do, respectively; statements about social norms asked whether they perceived other women in their community were doing these practices. Each statement was given a score of 1 for strongly agree or agree and 0 for strongly disagree, disagree, neither agree nor disagree. The knowledge, belief, social norms and self‐efficacy scores were constructed using outcomes‐specific factors, scaled and ranged from 0 to 10 and then on divided into high and low categories with cut‐offs at the median, for the regression analyses.

For household factors, support from husband and/or other family members was assessed by asking women whether husbands or other members helped to acquire diverse foods or IFA supplements, reminded them to consume them, monitored their weight and provided other support during pregnancy. Each statement was scored 1 for strongly agree or agree and 0 for strongly disagree, disagree, neither agree, nor disagree. The sum of scores was scaled and ranged from 0 to 10, and then divided into high and low categories with cut‐offs at the median. Measures of belief, self‐efficacy, perceived social norms and family support specifically related to breastfeeding were not collected due to study prioritization on maternal nutrition practices and limitations on length/duration of survey questionnaires.

Health service factors included timing of first ANC visit; number of ANC visits; home visit by a community health agent; receipt of IFA supplement for free and receipt of counselling or provision of information on dietary diversity, IFA supplementation, weight gain and breastfeeding.

### 2.5 Control variables

Control variables in these analyses included maternal age, education as a binary variable (no schooling/koranic literacy training/not completed first grade vs. primary/secondary or higher), religion (Muslim vs. Catholic/Protestant/Traditional) and parity (0 vs. 1, 2, 3+), which are common factors that influence dietary and other maternal nutrition practices. Household wealth index was constructed using principal components analysis of variables on housing conditions and assets, and the first component was used to divide the score into terciles (low vs. middle, high) (Vyas & Kumaranayake, 2006). Household food security was measured using the FANTA/USAID Household Food Insecurity Access Scale (Coates et al., 2007) and treated as a binary variable (food secure vs mildly/moderately/severely food insecure). For analyses of breastfeeding outcome, we included delivery at a health facility and caesarean section as control variables, as these influence the capacity for early breastfeeding practices.

### 2.6 Statistical analysis

Descriptive analysis was used to report the sample characteristics including the sociobehavioural factors. Bivariate analyses were used to test for associations between each potential determinant and the dependent variables. Multiple regression analyses were used to identify factors associated with MDD‐W during pregnancy (five or more food groups); consumption of at least 90 IFA tablets during the last pregnancy; weight monitoring at least 4 times during the last pregnancy; EIBF and EBF. Regression analyses were run adjusting for geographical clustering and control variables at maternal and household levels. Odds ratio (OR) with its 95% confidence interval was estimated for the logistic regression models. Population attributable risk is the proportion of the outcome in the population (exposed and unexposed) that is due to exposure. Thus, we used population attribute risk analysis (Newson, 2013) to estimate by how much our study outcomes can be improved under scenarios of either exposure to each modifiable determinant alone or in the combination of modifiable determinants, using the determinants identified from the regression results. From among the independent variables above, we considered modifiable factors as those that may be targeted and modified by programme interventions. Statistical significance levels at *p* < 0.05, *p* < 0.01 and *p* < 0.001 were used. All statistical analyses were performed using Stata version 17.

### 2.7 Ethical statement

Ethical approval was obtained from the Ethics Committee of Centre Muraz (Burkina Faso) and the Institutional Review Board of the International Food Policy Research Institute (USA). Written informed consent was obtained from all study participants.

## 3 RESULTS

### 3.1 Sample characteristics

The mean age of our study women was 27 years (ranged 15–48) (Table 1), and nearly all were married. About 66% of women had never attended school, and 7% received Koranic literacy training (Islamic learning system). Most women were Muslim (70%), and their main occupations were farmers (56%) or housewives (32%). Most pregnant women were in their second (44%) or third (41%) trimesters, and most women had three or more previous births (52% and 61%, respectively). One‐third of the study households were food insecure.

**Table 1:** Sample characteristics

Indicator	Pregnant women (*N* = 960)	Recently delivered women (*N* = 1920)
	Mean ± SD/Percent	Mean ± SD/Percent
Maternal factors		
Age (years)	27.4 ± 6.5	26.9 ± 6.7
Education level (%)		
Never attended school	66.0	65.5
Koranic literacy training	7.3	6.9
Primary school (grades 1–5)	17.0	16.0
Secondary school or higher (grades 6+)	9.7	11.6
Religion (%)		
Muslim	68.8	71.1
Catholic/Protestant	26.7	24.9
Traditional	4.4	4.0
Trimester of pregnancy (%)		
First (1–3 months)	15.2	‐
Second (4–6 months)	44.1	‐
Third (7months onward)	40.7	‐
Parity (%)		
0	15.3	‐
1	16.9	20.2
2	16.2	19.0
≥3	51.6	60.8
Knowledge of dietary diversity^a^ (*n*)	5.7 ± 2.3	5.9 ± 2.2
Knowledge of IFA^a^ (*n*)	5.2 ± 3.3	5.3 ± 3.3
Knowledge of weight gain during pregnancy (%)	6.5	10.7
Knowledge of breastfeeding^a^ (*n*)	‐	6.4 ± 1.9
Beliefs and self‐efficacy scores^a^ (*n*)	7.8 ± 1.7	7.7 ± 1.9
Perceived social norms scores^a^ (*n*)	6.0 ± 2.7	6.0 ± 2.6
Household factors		
Support from husband/family^a^ (*n*)	5.6 ± 3.6	6.2 ± 3.4
Household wealth (tertiles) (%)	33.3	33.3
Household food insecurity (%)	37.0	34.1
Health service factors		
Timing of first ANC visit (%)		
Early (≤3 months)	36.7	38.8
Intermediate (4–6 months)	41.9	55.4
Late (7–9 months)	1.5	5.8
4+ ANC visits (%)	‐	66.9
Visited at home by community health agent (%)	3.8	6.4
Received IFA tablet for free (%)	96.0	95.3
Received counselling on dietary diversity (%)	15.6	32.4
Received counselling on IFA (%)	76.5	94.9
Received counselling on weight gain (%)	4.0	7.1
Received counselling on breastfeeding (%)	12.3	27.0
Delivered at a health facility (%)	‐	92.2
Received caesarean section (%)	‐	2.4

Abbreviations: ANC, antenatal care; IFA, iron folic acid.

^a^ Scores for knowledge, beliefs and self‐efficacy, social norms and family support were rescaled and ranged from 0 to 10.

About one‐third of the study women had their first ANC visit during their first trimester of pregnancy, and 67% of recently delivered women completed four or more ANC visits. Although most recently delivered women received free IFA supplements (95%) and counselling on IFA (95%) during ANC, few received counselling about consuming a diverse diet (32%), adequate weight gain (7%) and breastfeeding (27%). Also, very few women received a home visit from a community health agent during the last pregnancy (6%).

### 3.2 Prevalence of maternal nutrition and early breastfeeding practices

**Figure 1:**
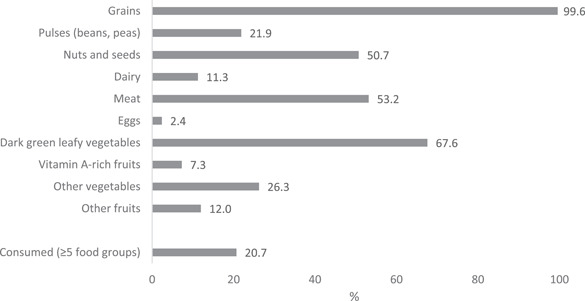
Proportion of pregnant women who consumed each of the 10 food groups and achieved minimum dietary diversity (≥ 5 food groups)

Nearly all pregnant women consumed grains, and more than half consumed dark green leafy vegetables (68%), meat (53%) and nuts and seeds (51%) in the past 24 h before the survey interview (Figure 1). Less than one‐quarter of the women had consumed pulses (22%), dairy (11%) or eggs (2%). Thus, only 21% of pregnant women achieved MDD‐W, that is, consumed at least five food groups.

**Figure 2:**
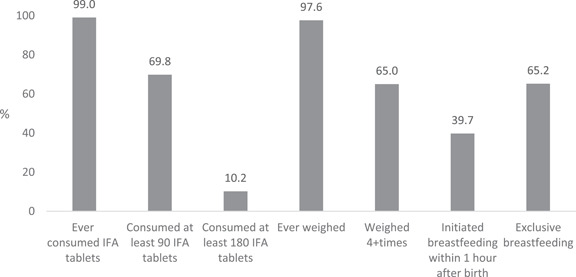
Prevalence of maternal nutrition and breastfeeding practices among recently delivery women. (1) Percentages reported are among all women. (2) Among all women, mean IFA tablets consumed and mean number of times weighed are 108 and 3.99, respectively.

Nearly all recently delivered women had consumed any IFA supplements during their last pregnancy, and 70% consumed at least 90 daily IFA tablets (Figure 2). However, only 10% of women consumed the total recommended amount of 180 IFA tablets during their last pregnancy. Nearly all women were weighed at least once during pregnancy, and 65% were weighed at least 4 times during their last pregnancy. In terms of early breastfeeding practices, 40% of women initiated breastfeeding within 1 h after birth, and 65% practised EBF in the first 6 months.

### 3.3 Determinants of dietary diversity among pregnant women

Maternal knowledge of dietary diversity was strongly associated with MDD‐W among *pregnant women* (Table 2). Those with high knowledge were 3.2 times more likely to consume at least 5 food groups, compared with women with low knowledge.

**Table 2:** Factors associated with minimum dietary diversity among pregnant women

	Bivariate		Multivariate	
Indicator	OR	95% CI	OR	95% CI
Maternal factors				
Knowledge of dietary diversity during pregnancy (ref: Low)				
High	2.56***	1.74, 3.78	3.24***	1.97, 5.34
Enabling beliefs and self‐efficacy about diet (ref: Low)				
High	0.96	0.66, 1.40	0.88	0.52, 1.48
Perceived social norms about diet (ref: Low)				
High	1.19	0.71, 1.97	1.77	0.91, 3.46
Household factors				
Support from husband/family member about diet (ref: Low)				
High	0.87	0.61, 1.26	0.77	0.46, 1.28
Health service factors				
Timing of ANC visits (ref: Intermediate to late)				
Early (≤3 months)	1.27	0.88, 1.84	1.16	0.76, 1.76
Visited at home by community health agent	1.29	0.67, 2.48	1.27	0.52. 3.06
Total ANC visits (ref: <4 ANC visits)				
4+ANC visits	0.99	0.67, 1.47	1.11	0.63, 1.94
Received counselling on dietary diversity	0.99	0.63, 1.58	0.77	0.42, 1.45
Control variables				
Maternal age (years)	1.00	0.98, 1.02	1.01	0.96, 1.06
Religion (ref: Others)				
Muslim	1.80**	1.16, 2.79	2.19**	1.23, 3.90
Education level (ref: No education)				
Primary school or higher	0.93	0.62, 1.40	1.16	0.69, 1.96
Parity (ref: 0)				
1	1.19	0.64, 2.20	1.63	0.66, 4.03
2	1.46	0.82, 2.60	2.20	0.85, 5.66
≥3	1.22	0.75, 2.00	1.58	0.61, 4.11
Household wealth (ref: Low)				
Intermediate	0.82	0.51, 1.29	0.79	0.47, 1.33
High	0.69	0.43, 1.11	0.62	0.34, 1.13
Household food security (ref: Insecure)				
Secure	1.23	0.87, 1.75	1.16	0.70, 1.93

Abbreviations: ANC, antenatal care; CI, confidence interval; OR, odds ratio.

** *p* < 0.01;

*** *p* < 0.001.

### 3.4 Determinants of IFA supplement consumption

Several maternal and health service factors were positively associated with the consumption of at least 90 IFA tablets during pregnancy (Table 3). Recently delivered women with high knowledge about IFA, positive perceived social norms about taking IFA supplements or support from husband or other family members were 1.5 times more likely to have consumed at least 90 IFA tablets during their last pregnancy, compared with low knowledge, low perceived social norms or low family support. Early initiation of ANC visit (first visit in the first trimester, compared with later trimesters) was associated with 1.5 times higher odds of consuming 90+ IFA tablets, and 4 or more ANC visits was associated with 10 times higher odds compared with less than 4 ANC visits. In addition, women who received counselling about IFA were 2.5 times more likely to consume 90+ IFA tablets. Similar patterns of positive association of high perceived social norms (OR: 2.1), high family support (OR: 1.9), early ANC (OR: 9.0) and 4 or more ANC visits (OR: 6.7) were observed with consumption of at least 180 IFA tablets during pregnancy (Supporting Information: Table S1).

**Table 3:** Factors associated IFA consumption (90+ tablets) among recently delivered women

	Bivariate		Multivariate	
Indicator	OR	95% CI	OR	95% CI
Maternal factors				
Knowledge of IFA (ref: Low)				
High	1.69***	1.32, 2.16	1.54*	1.04, 2.30
Enabling beliefs and self‐efficacy about IFA (ref: Low)				
High	1.26	0.98, 1.62	1.01	0.73, 1.40
Perceived social norms about IFA (ref: Low)				
High	1.62***	1.25, 2.10	1.52*	1.05, 2.21
Household factors				
Support from husband/family member about IFA (ref: Low)				
High	1.90***	1.50, 2.41	1.51*	1.00, 1.99
Health service factors				
Timing of ANC visits (ref: Intermediate to late)				
Early (≤3 months)	3.34***	2.41, 4.61	1.54*	1.03, 2.34
Visited at home by community health agent	1.75*	1.04, 2.95	1.10	0.49, 2.42
Total ANC visits (ref: <4 ANC visits)				
4+ANC visits	10.22***	7.41, 14.10	10.07***	7.05, 14.34
Received IFA supplements for free	1.36	0.68, 2.74	1.72	0.82, 3.58
Received counselling on IFA	2.22**	1.37, 3.61	2.48**	1.26, 4.90
Control variables				
Maternal age (years)	1.00	0.98, 1.01	1.00	0.97, 1.04
Religion (ref: Others)				
Muslim	0.68*	0.47, 0.96	0.67	0.43, 1.09
Education level (ref: No education)				
Primary or higher	1.01	0.79, 1.29	0.70	0.46, 1.05
Parity (ref: 1)				
2	0.81	0.58, 1.13	0.65	0.39, 1.08
≥3	0.80	0.62, 1.03	0.66	0.40, 1.09
Household wealth (ref: Low)				
Middle	0.99	0.71, 1.37	0.98	0.68, 1.41
High	0.97	0.69, 1.36	1.27	0.90, 1.79
Household food security (ref: Insecure)				
Secure	1.32*	1.04, 1.67	1.23	0.83, 1.82

Abbreviations: ANC, antenatal care; CI, confidence interval; IFA, iron folic acid; OR, odds ratio.

* *p* < 0.05;

** *p* < 0.01;

*** *p* < 0.001.

### 3.5 Determinants of weight monitoring

The number of ANC visits was highly correlated (*ρ*= 0.91) with the exposure to 4+ weighing during pregnancy; therefore, this variable was excluded from the regression models. Women who received early ANC visit (in the first trimester, compared with later trimesters) were 6.2 times more likely to have had their weight monitored at least 4 times during pregnancy, and those who received a home visit from a community health agent were also 2.4 times more likely to have received weight monitoring at least 4 times compared with women who received no home visit (Table 4).

**Table 4:** Factors associated with weight monitoring (weighed 4+ times) among recently delivered women

	Bivariate		Multivariate	
	OR	95%CI	OR	95% CI
Maternal factors				
Knowledge of weight gain (ref: Low)				
High	0.78	0.57, 1.10	0.88	0.60, 1.29
Perceived social norms about weight gain (ref: Low)				
High	0.94	0.75, 1.18	1.23	0.92, 1.66
Household factors				
Support from husband/family member about weight gain (ref: Low)				
High	1.40**	1.08, 1.80	1.14	0.82, 1.59
Health service factors				
Timing of ANC visits (ref: Intermediate to late)				
Early (≤3 months)	5.28***	3.98, 7.02	6.15***	4.24, 8.91
Visited at home by community health agent	2.13*	1.18, 3.84	2.36*	1.23, 4.53
Total ANC visits (ref: <4 ANC visits)^a^				
4+ ANC visits	‐	‐	‐	‐
Received counselling on weight gain	1.36	0.79, 2.34	0.95	0.47, 1.92
Control variables				
Maternal age (years)	1.00	0.99, 1.02	1.02	0.99, 1.04
Religion (ref: Others)				
Muslim	0.69*	0.52, 0.93	0.70*	0.52, 0.93
Education level (ref: No education)				
Primary school or higher	1.45**	1.13, 1.87	1.28	0.92, 1.77
Parity (ref: 1)				
2	1.11	0.81, 1.52	0.87	0.57, 1.32
≥3	0.90	0.70, 1.16	0.74	0.49, 1.11
Household wealth (ref: Low)				
Middle	1.01	0.75, 1.36	0.90	0.65, 1.25
High	0.67*	0.47, 0.96	0.68*	0.47, 0.99
Household food security (ref: Insecure)				
Secure	1.07	0.86, 1.35	1.24	0.88, 1.76

Abbreviations: ANC, antenatal care; CI, confidence interval; OR, odds ratio.

^a^ ANC visits highly correlated with outcome variable, *ρ* = 0.91.

* *p* < 0.05;

** *p* < 0.01;

*** *p* < 0.001.

### 3.6 Determinants of early initiation of breastfeeding

High maternal knowledge about breastfeeding was associated with 1.9 higher odds of EIBF in the first hour after birth (Table 5). Also, women who received breastfeeding counselling during ANC were 1.5 times more likely to have initiated breastfeeding within 1 h, compared with those who did not receive counselling about breastfeeding. Similar patterns of a positive association of high breastfeeding knowledge (OR: 3.3) and receipt of breastfeeding counselling (OR: 1.9) were observed for EBF (Supporting Information: Table S2).

**Table 5:** Factors associated with early initiation of breastfeeding among recently delivered women

	Bivariate		Multivariate	
	OR	95% CI	OR	95% CI
Maternal factors				
Knowledge of breastfeeding (ref: Low)				
High	223***	1.78, 2.80	1.90***	1.42, 2.54
Health service factors				
Timing of ANC visits (ref: Intermediate to late)				
Early (≤3 months)	1.23*	1.01, 1.51	1.14	0.88, 1.47
Visited at home by community health agent	1.21	0.76, 1.94	0.86	0.50, 1.52
Total ANC visits (ref: <4 ANC visits)				
4+ ANC visits	1.47**	1.15, 1.88	1.28	0.92, 1.79
Received counselling on breastfeeding	1.64**	1.23, 2.19	1.49*	1.06, 2.10
Control variables				
Maternal age (years)	1.00	0.99, 1.02	0.99	0.96, 1.02
Religion (ref: Others)				
Muslim	0.75*	0.57, 0.98	0.74	0.53, 1.06
Education level (ref: No education)				
Primary school or higher	0.99	0.80, 1.23	0.94	0.70, 1.26
Parity (ref: 1)				
2	1.34	0.99, 1.82	1.38	0.90, 2.13
≥3	1.11	0.86, 1.43	1.12	0.70, 1.79
Child age (months)	1.03	0.97, 1.08	1.02	0.95, 1.09
Child sex as male	1.18	0.99, 1.40	1.24	1.00, 1.55
Delivered by c‐section	0.55	0.27, 1.11	0.75	0.30, 1.85
Household wealth (ref: Low)				
Middle	1.11	0.82, 1.51	1.08	0.78, 1.48
High	1.01	0.69, 1.49	1.11	0.76, 1.61
Household food security (ref: insecure)				
Secure	1.18	0.90, 1.56	1.12	0.79, 1.60

* *p* < 0.05;

** *p* < 0.01;

*** *p* < 0.001.

### 3.7 Population attributable risk estimation of modifiable factors

Select maternal, household and health service factors were significantly positively associated with dietary diversity, consumption of IFA supplements, weight monitoring and early breastfeeding practices (Tables 3, 4, 5). Those identified modifiable factors were used in the population attributable risk analyses to estimate how much the outcome practices could be improved by increasing the determinants among all the women (optimal scenarios achieved by interventions) compared with the current baseline levels.

**Figure 3:**
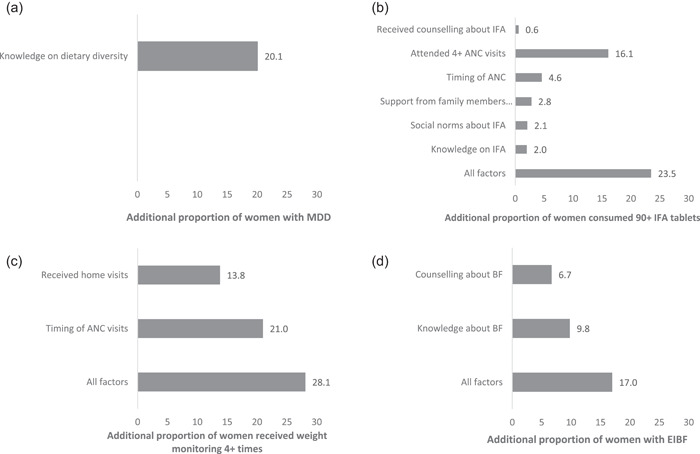
Population attributable risk estimation by outcome. (a) Minimum dietary diversity; (b) Consumption of 90+ IFA tablets; (c) Weighed 4+ times; (d) Early initiation of breastfeeding.

If all pregnant women obtained high knowledge of dietary diversity, we estimated a 20% increase in MDD‐W (Figure 3a). Given that 21% of women currently consumed at least 5 food groups, this would result in a total of 41% of pregnant women achieving MDD‐W. Under the combined conditions of high IFA knowledge, high perceived social norms, high family support, early ANC, 4 or more ANC visits and IFA counselling, we estimated a 24% increase in the consumption of at least 90 IFA tablets during pregnancy, resulting in an overall total of 93% (Figure 3b). Under the combined conditions, there would also be a 25% increase in the consumption of at least 180 IFA tablets, resulting in a total of 35% (Supporting Information: Figure S1). With early ANC initiation and exposure to home visits, an increase of 28% in weight monitoring at least 4 times is estimated, resulting in a total of 93% (Figure 3c). Under the conditions of high breastfeeding knowledge and breastfeeding counselling, an increase of 17% in EIBF is estimated, resulting in a total of 57% (Figure 3d). Under these combined conditions, a 21% increase is estimated for a total of 86% in EBF (Supporting Information: Figure S2).

## 4 DISCUSSION

There are clearly factors modifiable through interventions at the individual, household and health service levels for improving maternal nutrition and breastfeeding practices. Our study showed that these modifiable determinants are outcome‐specific, that is, practices were associated with different sets of factors. High maternal knowledge was an important factor for MDD‐W, breastfeeding and IFA consumption. Family support and positive social norms were influential on the consumption of 90+ IFA tablets. Health service factors such as timing of first ANC visit, 4+ ANC visits, receipt of home visits and counselling were important factors for IFA consumption, weight monitoring and breastfeeding.

Assuring minimally adequate diet diversity, classified using MDD‐W which was shown to be positively associated with adequate micronutrient intakes among pregnant women in rural Burkina Faso (Diop et al., 2021), is critical, but achieved by only one‐fifth of women in our study. The proportion of pregnant women with MDD‐W in our sample (21%) was slightly lower than the 28% among women of reproductive age living in the urban area of Bobo‐Dioulasso (capital of one of our study regions) (Custodio et al., 2020); thus, there was a large potential to improve this practice across both rural and urban areas. A previous study in a rural community in Burkina Faso also observed insufficient nutrient intakes among pregnant women and no major differences in food intake compared with nonpregnant women, so the additional nutritional needs of pregnancy are not accounted for in their dietary practices (Huybregts et al., 2009). Among the various determinants of dietary diversity during pregnancy examined, however, high knowledge only was a strongly associated factor observed in our study. In a maternal nutrition study in Bangladesh, knowledge about adequate diet (OR: 1.8) was observed to be associated with MDD‐W among pregnant women, as well as combined behavioural determinants (positive beliefs, self‐efficacy and social norms; OR: 1.8) and high husband support (OR: 1.9) (Nguyen et al., 2017). In India, high knowledge (OR: 2.2) and receipt of counselling on dietary diversity (OR: 1.9) were associated with MDD‐W (Nguyen et al., 2019). Our study finding on the importance of knowledge corroborated the results common in these previous studies, while we also acknowledge that knowledge is necessary but is not sufficient for women to achieve optimal nutrition practices. Although counselling may have been hypothesized to be a determinant in our study, the low exposure to counselling about diet dietary during pregnancy and potentially low quality of counselling (Nikiema et al., 2010) may have contributed to their weak influence, and women's knowledge about diet may have been obtained from sources outside of counselling during ANC. Furthermore, we measured counselling receipt but not quality of counselling in terms of interpersonal communication and respectful care, which are more difficult to assess.

Consumption of any IFA supplements during pregnancy was ubiquitous, but there was a 30% point decrease among recently delivered women consuming a 3‐month supply (90+ tablets), and a more dramatic drop‐off in achieving the recommended consumption period of 6 months (180+ tablets). However, various modifiable maternal, household and health service factors were observed to be associated with the consumption of 90+ IFA tablets, including high IFA knowledge, positive social norms about IFA, early ANC initiation, 4+ ANC visits and counselling on IFA. Our results are similar to the previous study results in Bangladesh and India (Nguyen et al., 2019). Above all, we observed that 4+ ANC visits were associated with 10 times higher odds, followed by counselling on IFA with 2.5 higher odds of consuming 90+ IFA tablets. The influence of a high number of ANC visits was not surprising, given that the local health centre was the main source for IFA supplements for women in our study (98% of pregnant women reported the health centre as their primary source). For achieving consumption of 180+ IFA tablets, early ANC initiation and 4+ ANC visits were the most prominent determinants. The factors associated with adherence to IFA supplementation during pregnancy has have been examined by numerous studies around the world. Recent studies from even a few African countries observed that ANC timing, anaemia knowledge, counselling on IFA and distance to health facility were important factors for adherence in Tanzania (Lyoba et al., 2020); knowledge about anaemia and benefits of IFA and health education were important in Ethiopia (Assefa et al., 2019) and ANC attendance and support from husband were observed to be influential in Niger (Begum et al., 2018). Our findings corroborate the factors identified in many of these previous studies.

Inasmuch as the WHO guidelines for ANC recommends counselling on healthy eating and physical activity during pregnancy to maintain adequate weight gain (World Health Organization, 2016), monitoring of weight gain during pregnancy is a critical step towards achieving this recommendation. Although weight gain monitoring involves tracking the additional weight gained between different gestational periods and comparing progress to recommended ranges to healthy weight gain, we assessed weight monitoring as simply weight measured and recorded during last pregnancy, as less than 14% of women in our study did not receive any counselling about weight gain. Weight monitoring during pregnancy was nearly universal in our study area, and 65% of women were weighed 4+ times during last pregnancy. Weighed 4+ times was highly correlated with 4+ ANC visits, so weight measurement appeared to take place usually at each ANC visit. Aside from the number of ANC visits, early initiation of ANC and receipt of home visit by community health agents were highly associated with being weighted 4+ times during pregnancy, as they likely increased the opportunity period for being weighed and promoted ANC visits where weight monitoring takes place. Our results were overlapped on two out of five modifiable factors identified in the maternal nutrition study in India (i.e., knowledge about weight gain, family support, early timing of ANC, 4+ ANC and counselling on weight gain were associated with the number of times weighed) (Nguyen et al., 2019).

EIBF (40%) and EBF (65%) prevalence in our study were similar (for EIBF) and higher (for EBF) than nationally representative levels (42% and 38%, respectively) (INSD & ICF International, 2012; Victora et al., 2016) and substantially higher than EBF (30%) reported in a previous A&T breastfeeding study before interventions in Boucle du Mouhoun (Cresswell et al., 2017), which is one of our study regions. There was still large room for improvement of both practices, particularly for EIBF. Predictors of EIBF and EBF have also been studied extensively in various settings; a recent study of 13 West African countries observed that caesarean delivery, no ANC visits, nonskilled delivery were negatively associated with EIBF (Ezeh et al., 2019), and predictors of EBF identified in the Burkina Faso study included younger infant age, institutional birth, postnatal care attendance and EBF knowledge (Cresswell et al., 2017). Our findings of breastfeeding knowledge and counselling as important modifiable factors for EIBF and EBF were among the key factors identified at the individual and health service levels (Cresswell et al., 2017; Rollins et al., 2016).

Our study examined multiple levels of factors that influence essential maternal nutrition and breastfeeding practices. By modelling the associated factors that may be modified by programme interventions, we observed that almost all the women may be able to achieve consumption of 90+ IFA tablets and receipt of weight monitoring 4+ times under optimal conditions, and nearly half or above half of the women may be able to achieve MDD‐W and EIBF. These findings and scenarios present the potential opportunities that may be achieved through interventions such as quality nutrition counselling/education and enhanced ANC services that address these modifiable factors.

A study limitation is related to the cross‐sectional design, from which we examined associations between determinants and outcomes but cannot draw conclusions on causality. Although we measured various outcome‐specific factors at the maternal, household and health service levels, we were limited in our survey to collect exhaustive data on all factors; thus, some psychosocial factors specifically related to breastfeeding (i.e., belief and self‐efficacy, social norms and family support) were not available and excluded in our analyses for EIBF and EBF. Lastly, maternal nutrition and breastfeeding practices were self‐reported by women and may be subject to recall bias and social desirability bias. However, we corroborated our baseline levels with other data sources wherever feasible.

Our study provided evidence on a range of determinants that may be modified by interventions to improve maternal nutrition and breastfeeding practices in Burkina Faso. Strengthening the delivery and uptake of interventions targeted at modifiable factors has the potential to improve maternal nutrition during pregnancy and early breastfeeding practices.

## AUTHOR CONTRIBUTIONS

Sunny S. Kim, Césaire T. Ouédraogo and Purnima Menon designed the study. Rasmané Ganaba, Maurice G. Zafimanjaka and Manisha Tharaney contributed to the implementation of the study. Césaire T. Ouédraogo and Rock R. Zagré conducted analyses. Sunny S. Kim and Césaire T. Ouédraogo wrote the first draft of the manuscript, and all authors read and approved the final manuscript.

## CONFLICT OF INTEREST

The authors declare no conflict of interest.

## Supporting information

Supplementary information.

## Data Availability

The data that support the findings of this study are available in Harvard Dataverse at https://doi.org/10.7910/DVN/LKI25D and https://doi.org/10.7910/DVN/Q09GCR.
