# Supplementary material for: Multiple modifiable maternal, household and health service factors are associated with maternal nutrition and early breastfeeding practices in Burkina Faso
Source: Matern Child Nutr. 2022 Nov 14;19(1):e13457. doi: 10.1111/mcn.13457 (PMC9749589; doi:10.1111/mcn.13457)
Supplement: Supplementary file 1 — Supplementary information. [file MCN-19-e13457-s001.docx]

**Table S1. Factors associated IFA consumption (180+ tablets) among recently delivered women**

|  | **Bivariate** | | **Multivariate** | |
| --- | --- | --- | --- | --- |
|  | **OR** | **95%CI** | **OR** | **95%CI** |
| **Maternal factors:** |  |  |  |  |
| Knowledge of IFA (ref: low) |  |  |  |  |
| High | 1.19* | 0.76, 1.86 | 0.82 | 0.44, 1.52 |
| Enabling beliefs and self-efficacy about IFA (ref: low) |  |  |  |  |
| High | 1.48* | 1.01, 2.17 | 1.26 | 0.76, 2.08 |
| Perceived social norms about IFA (ref: low) |  |  |  |  |
| High | 1.67* | 1.12, 2.49 | 2.14** | 1.30, 3.51 |
| **Household factors:** |  |  |  |  |
| Support from husband/family member about IFA (ref: low) |  |  |  |  |
| High | 2.14*** | 1.51, 3.03 | 1.92* | 1.13, 3.26 |
| **Health service factors:** |  |  |  |  |
| Timing of ANC visits (ref: intermediate to late) |  |  |  |  |
| Early (≤3 months) | 10.93*** | 6.94, 17.22 | 9.02*** | 5.02, 16.22 |
| Visited at home by community health agent | 1.64 | 0.85, 3.16 | 1.31 | 0.64, 2.70 |
| Total ANC visits (ref: <4 ANC visits) |  |  |  |  |
| 4+ANC visits | 12.72*** | 6.02, 26.89 | 6.68** | 2.23, 20.00 |
| Received IFA for free | 1.00 | 0.44, 2.30 | 0.56 | 0.20, 1.58 |
| Received counselling on IFA | 1.14 | 0.44, 2.92 | 2.26 | 0.41, 12.26 |
| **Control variables:** |  |  |  |  |
| Maternal age (years) | 1.00 | 0.98, 1.02 | 1.05* | 1.01, 1.10 |
| Religion (ref: others) |  |  |  |  |
| Muslim | 1.09 | 0.74, 1.62 | 0.91 | 0.52, 1.60 |
| Education level (ref: no education) |  |  |  |  |
| Primary school or higher | 1.04 | 0.67, 1.60 | 0.72 | 0.34, 1.54 |
| Parity (ref: 1) |  |  |  |  |
| 2 | 0.74 | 0.46, 1.18 | 0.56 | 0.30, 1.06 |
| ≥3 | 0.67* | 0.49, 0.91 | 0.30** | 0.15, 0.59 |
| Household wealth (ref: low) |  |  |  |  |
| Middle | 0.91 | 0.61, 1.36 | 0.82 | 0.52, 1.29 |
| High | 0.72 | 0.40, 1.32 | 0.91 | 0.49, 1.68 |
| Household food security (ref: insecure) |  |  |  |  |
| Secure | 0.95 | 0.67, 1.35 | 0.93 | 0.53, 1.61 |

*p<0.05; **p<0.01;***p<0.001

**Figure S1. Population attributable risk estimation of select modifiable factors on IFA consumption (180+ tablets)**

**Table S2. Factors associated with exclusive breastfeeding**

|  | **Bivariate** | | **Multivariate** | | |  |
| --- | --- | --- | --- | --- | --- | --- |
|  | **OR** | **95%CI** | | **OR** | **95%CI** | |
| **Maternal factors:** |  |  | |  |  | |
| Knowledge of breastfeeding (ref: Low) |  |  | |  |  | |
| High | 3.43*** | 2.64, 4.43 | | 3.32*** | 2.36, 4.67 | |
| Health service factors: |  |  | |  |  | |
| Timing of ANC visits (ref: Intermediate to late) |  |  | |  |  | |
| Early (≤3 months) | 1.29* | 1.04, 1.60 | | 1.09 | 0.78, 1.52 | |
| Visited at home by community health agent | 1.19 | 0.76, 1.87 | | 0.98 | 0.59, 1.68 | |
| Total ANC visits (ref: <4 ANC visits) |  |  | |  |  | |
| 4+ ANC visits | 1.41** | 1.11, 1.80 | | 1.25 | 0.86, 1.80 | |
| Received counselling on breastfeeding | 1.84*** | 1.31, 2.59 | | 1.87** | 1.18, 2.94 | |
| **Control variables:** |  |  | |  |  | |
| Maternal age (years) | 1.01 | 1.00, 1.03 | | 0.96** | 0.94, 0.99 | |
| Religion (ref: Others) |  |  | |  |  | |
| Muslim | 0.66** | 0.49, 0.87 | | 0.63** | 0.46, 0.86 | |
| Education level (ref: No education) |  |  | |  |  | |
| Primary school or higher | 0.82 | 0.66, 1.00 | | 0.76 | 0.56, 1.04 | |
| Parity (ref: 1) |  |  | |  |  | |
| 2 | 1.89*** | 1.43, 2.51 | | 1.73** | 1.16, 2.57 | |
| ≥3 | 1.74*** | 1.37, 2.21 | | 2.18*** | 1.41, 3.35 | |
| Child age (months) | 0.75*** | 0.70, 0.80 | | 0.70*** | 0.64, 0.76 | |
| Child sex as male | 1.07 | 0.90, 1.27 | | 0.99 | 0.76, 1.29 | |
| Household wealth (ref: Low) |  |  | |  |  | |
| Middle | 1.04 | 0.78, 1.39 | | 1.08 | 0.78, 1.49 | |
| High | 1.19 | 0.88, 1.62 | | 1.37 | 0.98, 1.88 | |
| Household food security (ref: Insecure) |  |  | |  |  | |
| Secure | 1.28* | 1.01, 1.63 | | 1.04 | 0.78, 1.41 | |

*p<0.05; **p<0.01;***p<0.001

**Figure S2. Population attributable risk estimation of select modifiable factors on exclusive breastfeeding**
